# Supplementary material for: Advancing the science of dynamic airborne nanosized particles using Nano-DIHM
Source: Commun Chem. 2021 Dec 8;4:170. doi: 10.1038/s42004-021-00609-9 (PMC9814397; doi:10.1038/s42004-021-00609-9)
Supplement: Supplementary file 2 — Supplementary Information [file 42004_2021_609_MOESM2_ESM.pdf]

Advancing the Science of Dynamic Airborne Nanosized Particles using Nano-DIHM

Devendra Pal[1], Yevgen Nazarenko[1], Thomas C. Preston[1,2], and Parisa A. Ariya[*,1,2]

[1] Department of Atmospheric and Oceanic Sciences, McGill University, 805 Sherbrooke Street West, Montreal, QC H3A 0B9, Canada;

[2] Department of Chemistry, McGill University, 801 Sherbrooke Street West, Montréal, QC H3A 2K6, Canada.

*Corresponding author: Parisa A. Ariya

Phone: (514) 398-6931 & (514) 398-3615

Fax: (514) 398-3797

E-mail: parisa.ariya@mcgill.ca

Table of Contents

Table of Contents 2

Supplementary Figures 3

Supplementary Tables 17

Supplementary Notes ………………………………………………………………………..19

Supplementary Figures

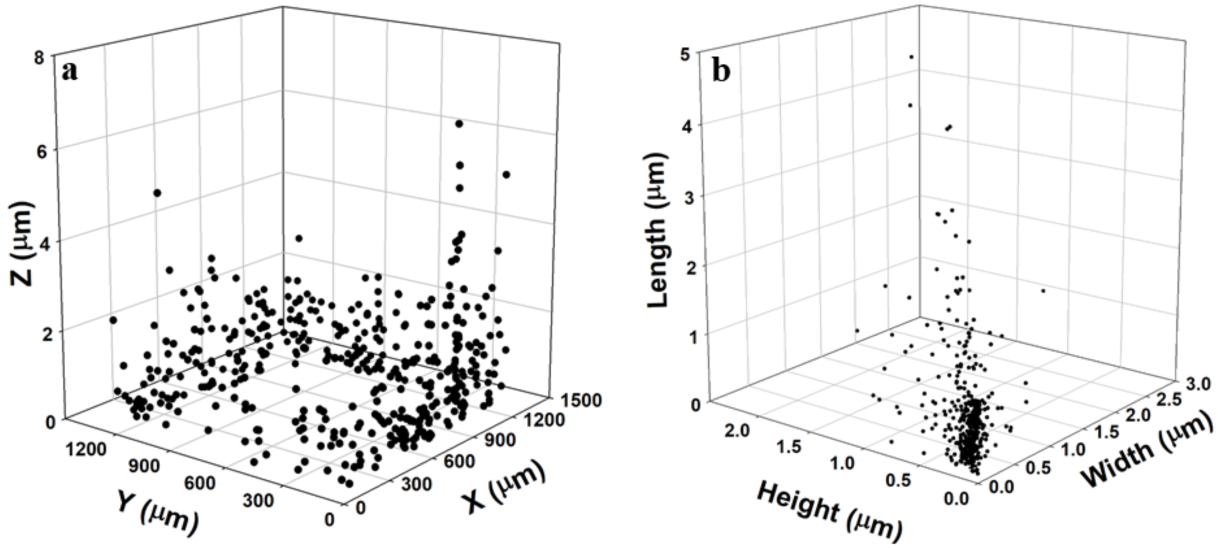

c

Statistics	Width (µm)	Height (µm)	Length (µm)
Mean values	0.265	0.265	0.748
Standard deviation	0.290	0.276	0.663
Median values	0.176	0.176	0.644
99th percentile	1.374	1.326	3.791
1st percentile	0.014	0.014	0.013

Figure S1 3D size distribution of 100 nm, PSL spheres in the aerosol phase. (a) The orientation of PSL particles (b) Width, height, and length distribution of PSL particles in a single hologram over 62.5 ms. (c) Tabular representation of descriptive statistics of the distributions of dimensions of the PSL spheres in 3D space in a single hologram. The results shows the ± 5% variation from one hologram to another.

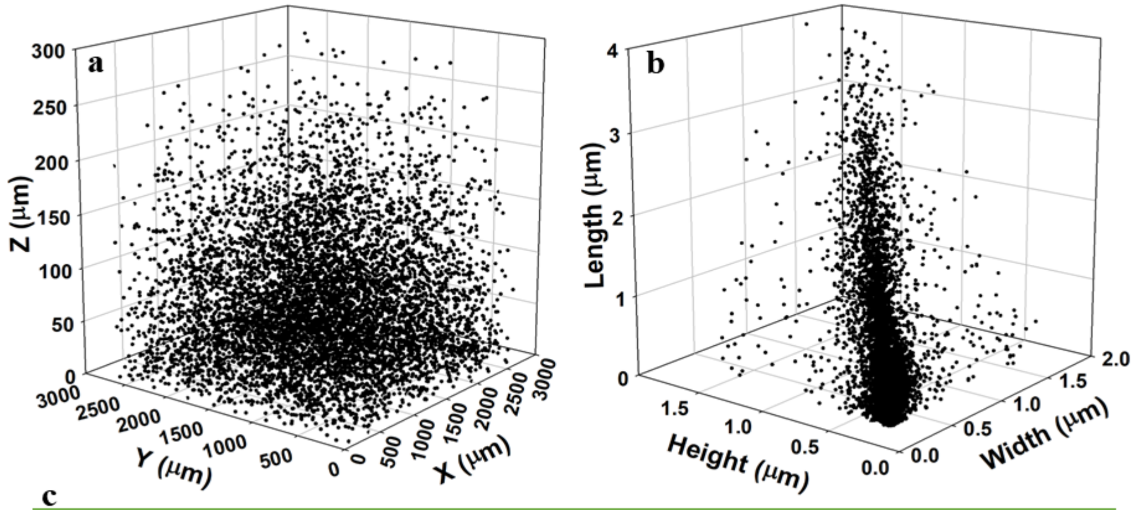

c

Statistics	Width (µm)	Height (µm)	Length (µm)
Mean values	0.401	0.394	0.794
Standard deviation	0.320	0.311	0.639
Median values	0.319	0.319	0.678
99th percentile	1.420	1.345	3.109
1st percentile	0.023	0.023	0.015

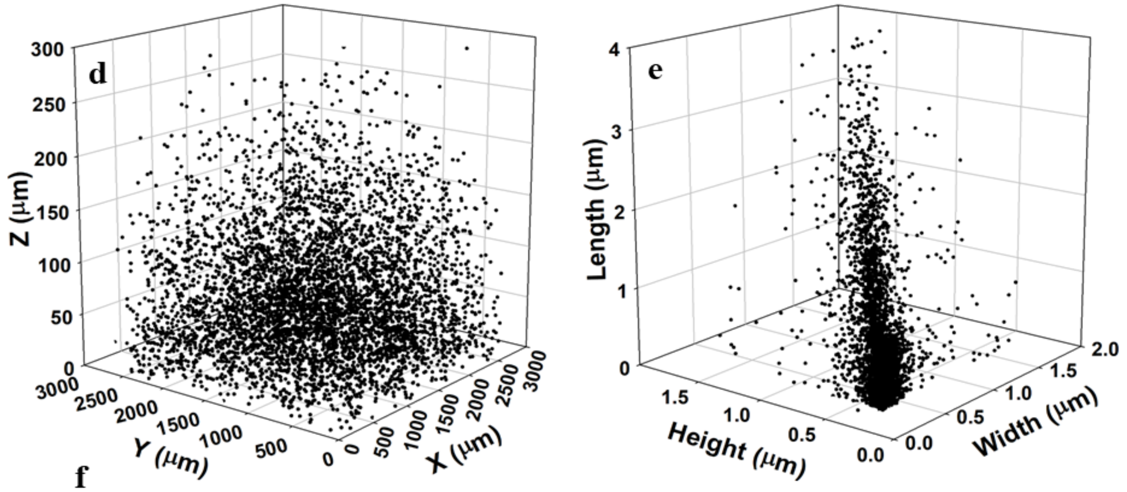

f

Statistics	Width (µm)	Height (µm)	Length (µm)
Mean values	0.381	0.378	0.805
Standard deviation	0.303	0.299	0.593
Median values	0.302	0.303	0.728
99th percentile	1.335	1.287	2.960
1st percentile	0.023	0.022	0.007

Figure S2 3D size distribution of 200 nm, PSL spheres in the aerosol phase. (a) The orientation of PSL particles (b) Width, height, and length distribution of PSL particles in a single hologram. (c) Tabular representation of descriptive statistics of the distributions of dimensions of the PSL spheres in 3D space in a single hologram. (d-f) 3D size distribution of 200 nm PSL different hologram. The results are very consistent, and the median dimension of PSL varies within 5%.

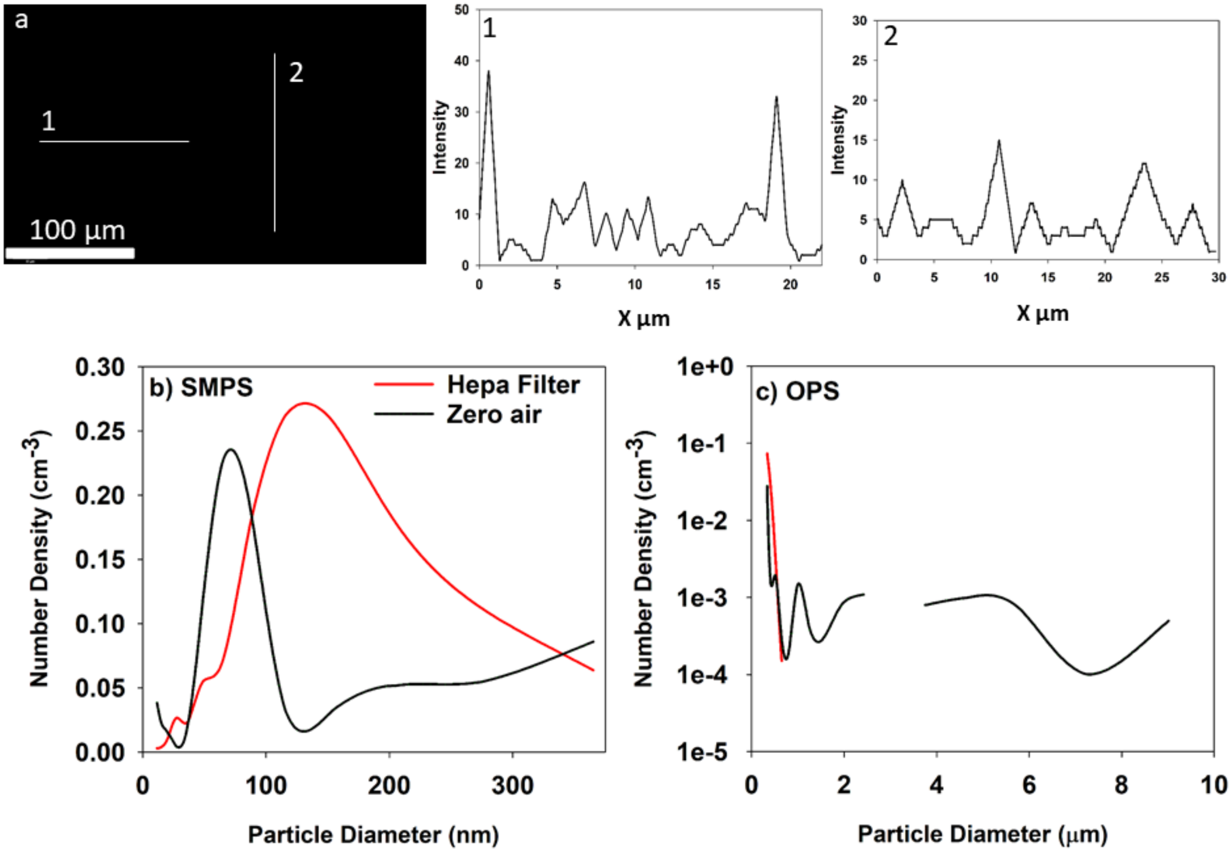

Figure S3 Background intensity holograms (a) zero air (crosscut size plotted with horizontal line 1 and vertical line 2. (b, c) particle size distribution of zero air and the air filter with a HEPA filter.

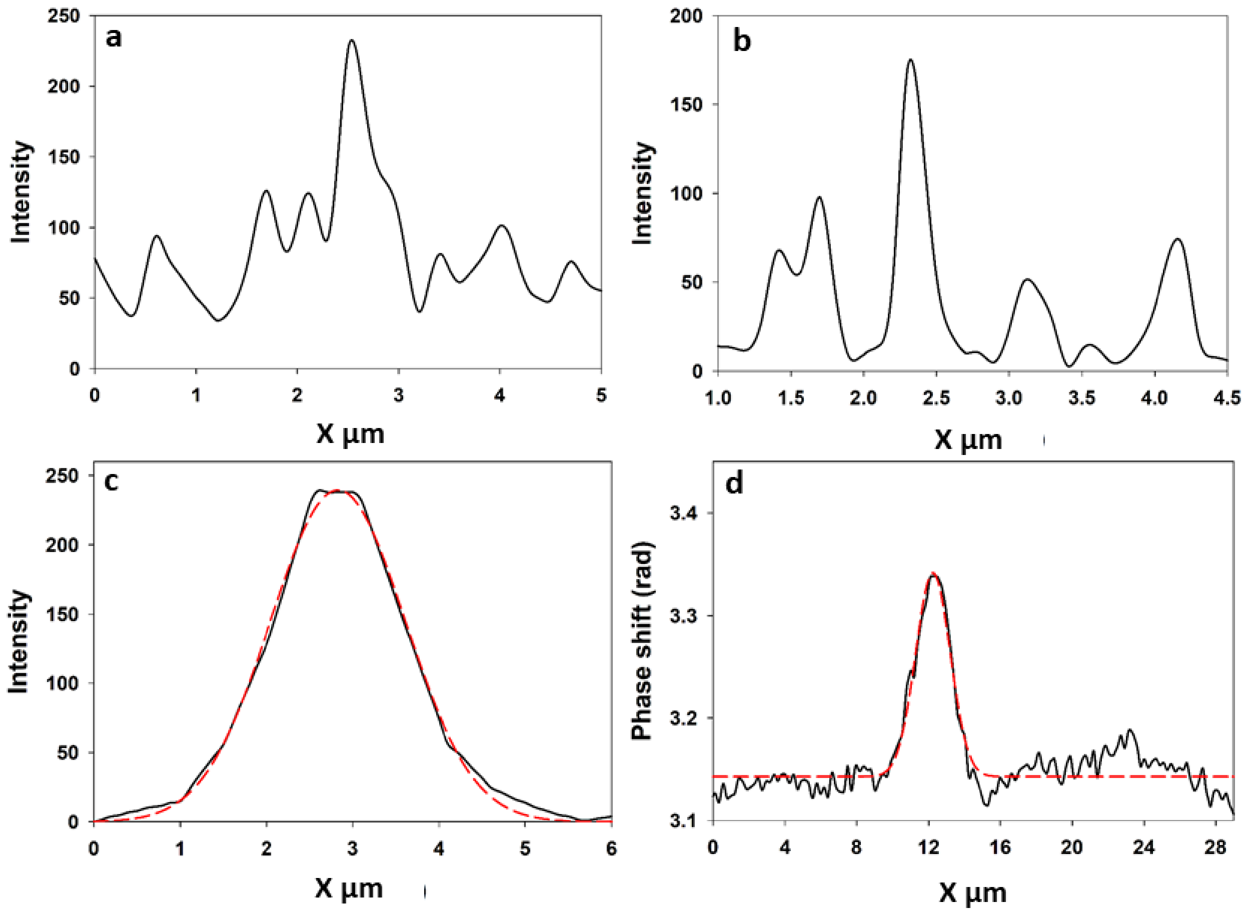

Figure S4 Reconstruction of the intensity and phase images for PSL spheres in the moving air. Single particles and small clusters of PSL particles in different positions inside the cuvette. (a-c) Intensity profile of PSL particles across the particle crosscut. (d) Phase profile through a single-particle crosscut (same particle as in ‘c’). The red long-dash curve in (c-d) is a dynamic Gaussian fitting, which suggests a perfect fitting with the experimental results of a single PSL sphere.

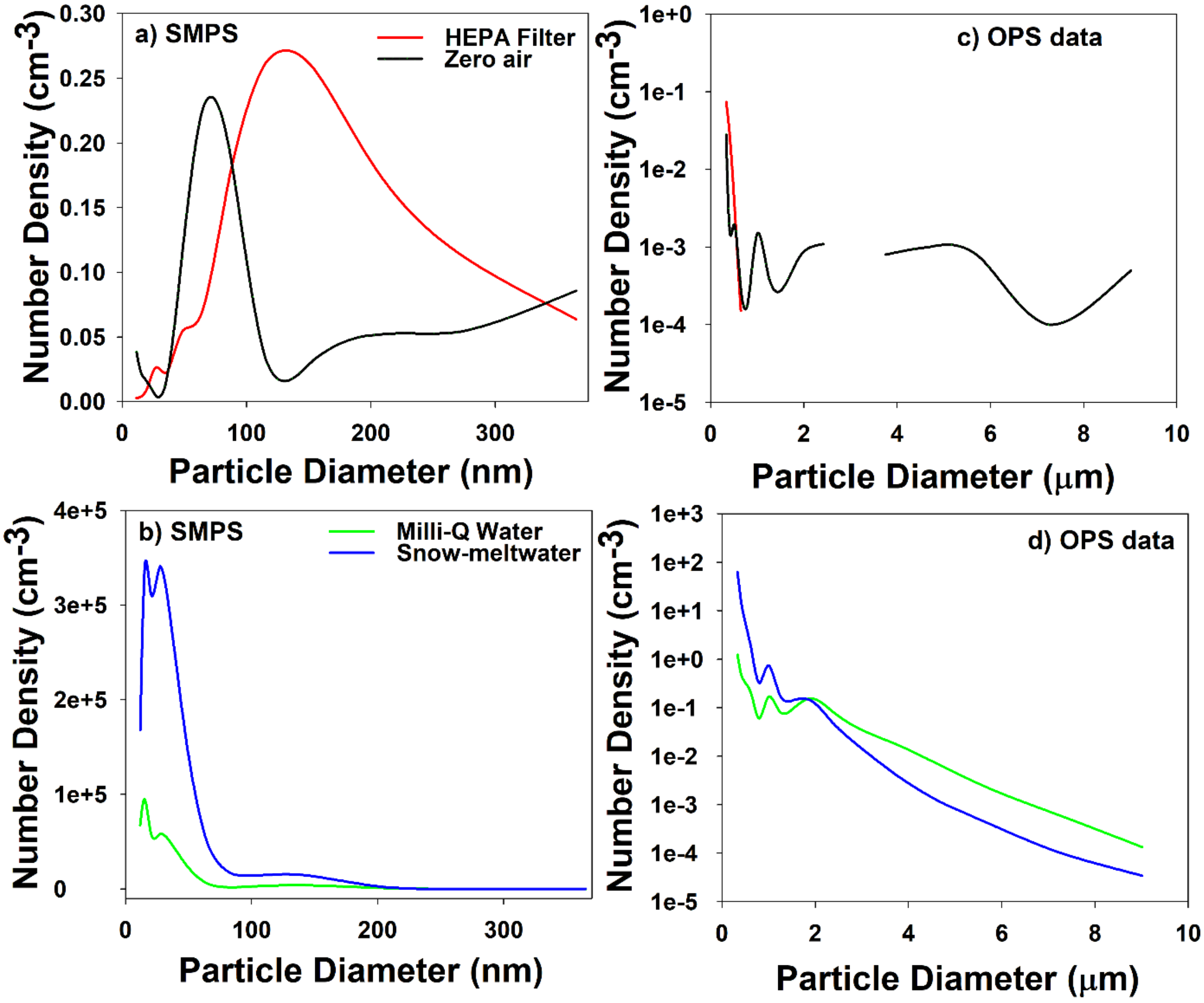

Figure S5 Particle size distribution of Zero air; air passes through a HEPA filter and snow-borne particles in moving air (aerosolized snow meltwater). (a, b) Particle size distribution in the range from 10 nm to 350 nm measured by the scanning mobility particle sizer (SMPS). (c, d) Particle size distribution in the range from 0.3 µm to 10 µm, measured by the optical particle sizer (OPS).

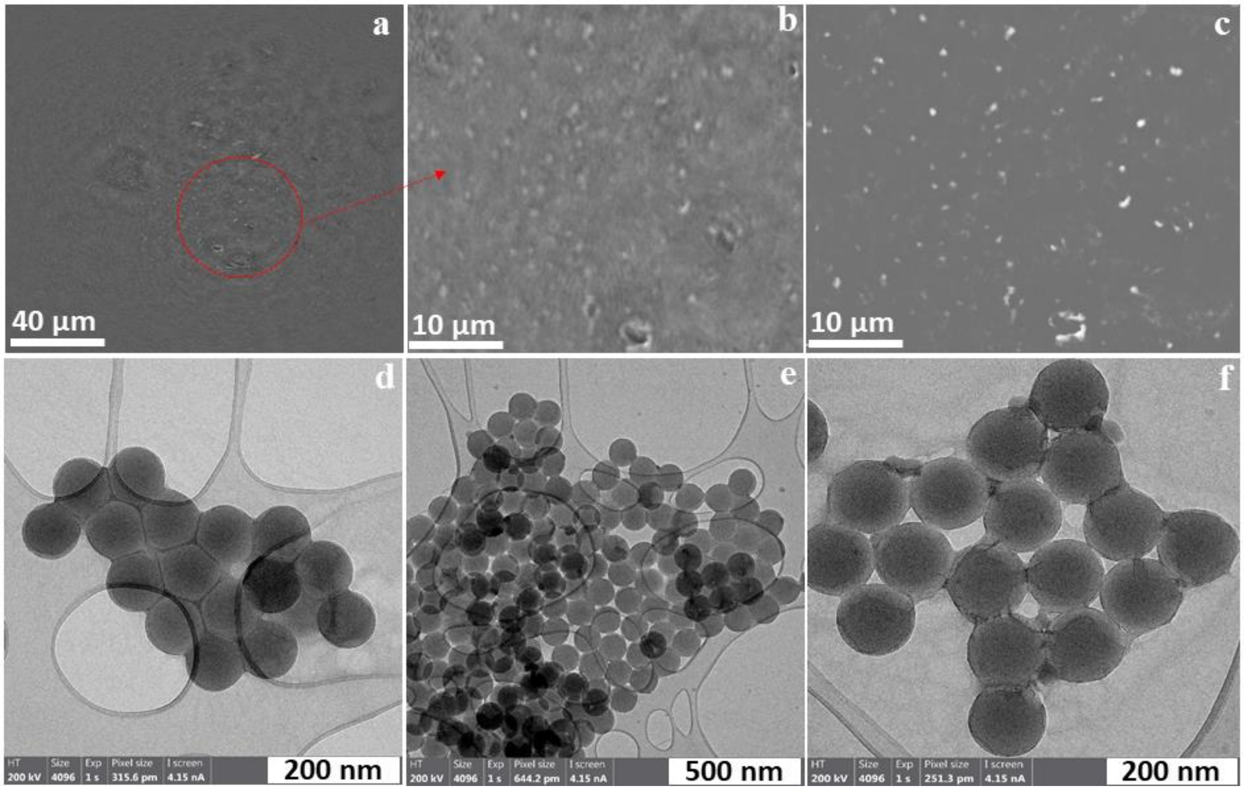

Figure S6 Holographic reconstruction of 200 nm PSL particles deposited on microscopy slide and their validation by STEM images. (a) Phase reconstruction at 2352 µm. (b) Zoomed in area of (a). (c) In focus high-resolution reconstruction at Z=2419 µm (d-f) S/TEM images of same 200 nm PSL samples.

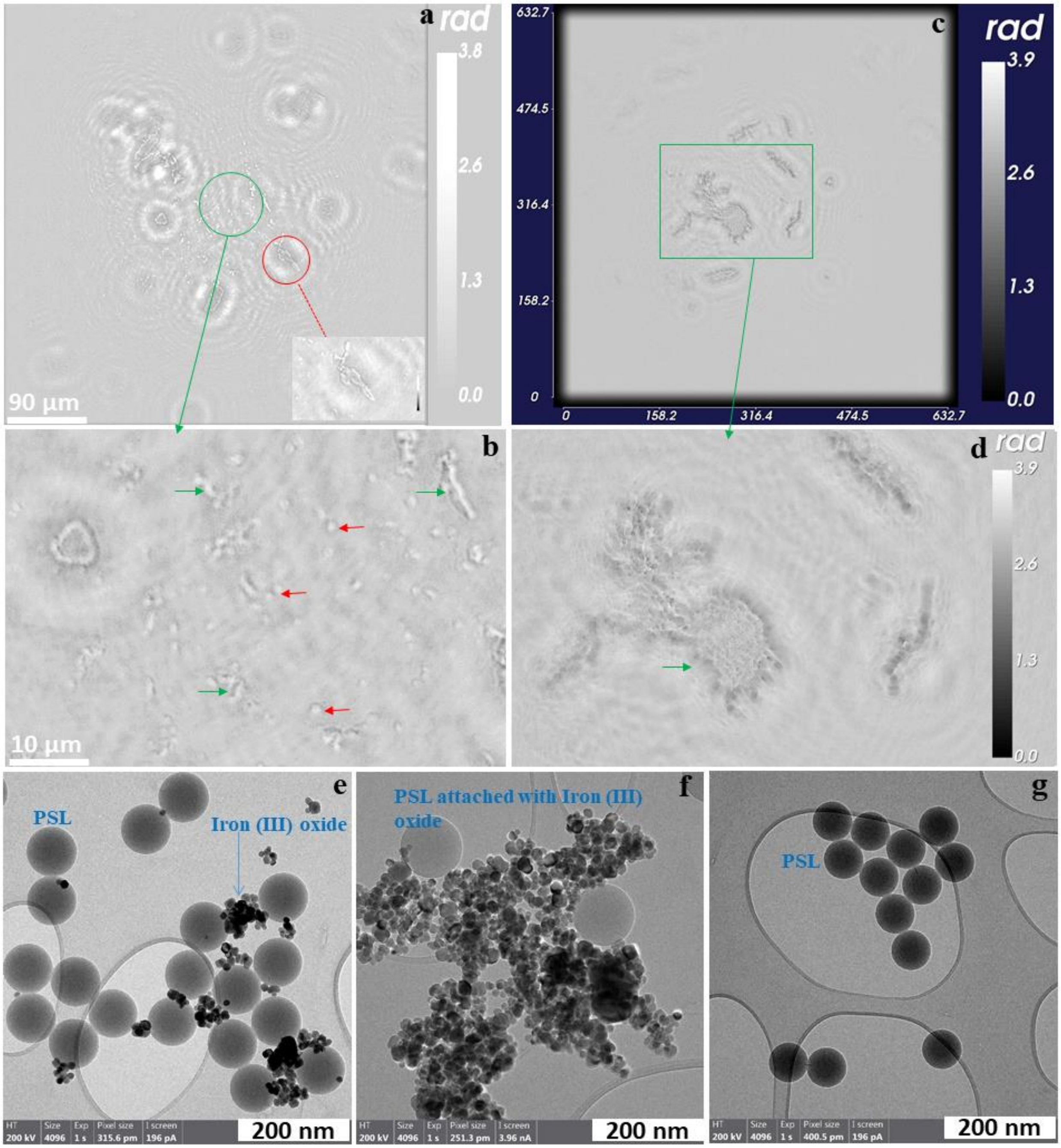

Figure S7 2D and 3D view of holographic reconstruction of a mixed sample of 200 nm PSL particles and iron(III) oxide nanoparticles (size <50 nm) deposited on microscopy slide and their validation by S/TEM images. (a) 2D view of phase reconstruction at 925 µm. (b) Zoomed in area circle (a). The red arrow in figure (b) displayed the PSL particles while green arrow shows attachment of PSL on iron particles (c) 3D view of phase reconstruction at 562 µm. (d) Zoomed in area ranctangle shows in figure (c). (e-f) S/TEM images of the same sample confirmed the existence of both

PSL and iron oxide nanoparticles. 3D view of phase reconstruction clearly indicates the attchement of PSL on iron oxide particles.

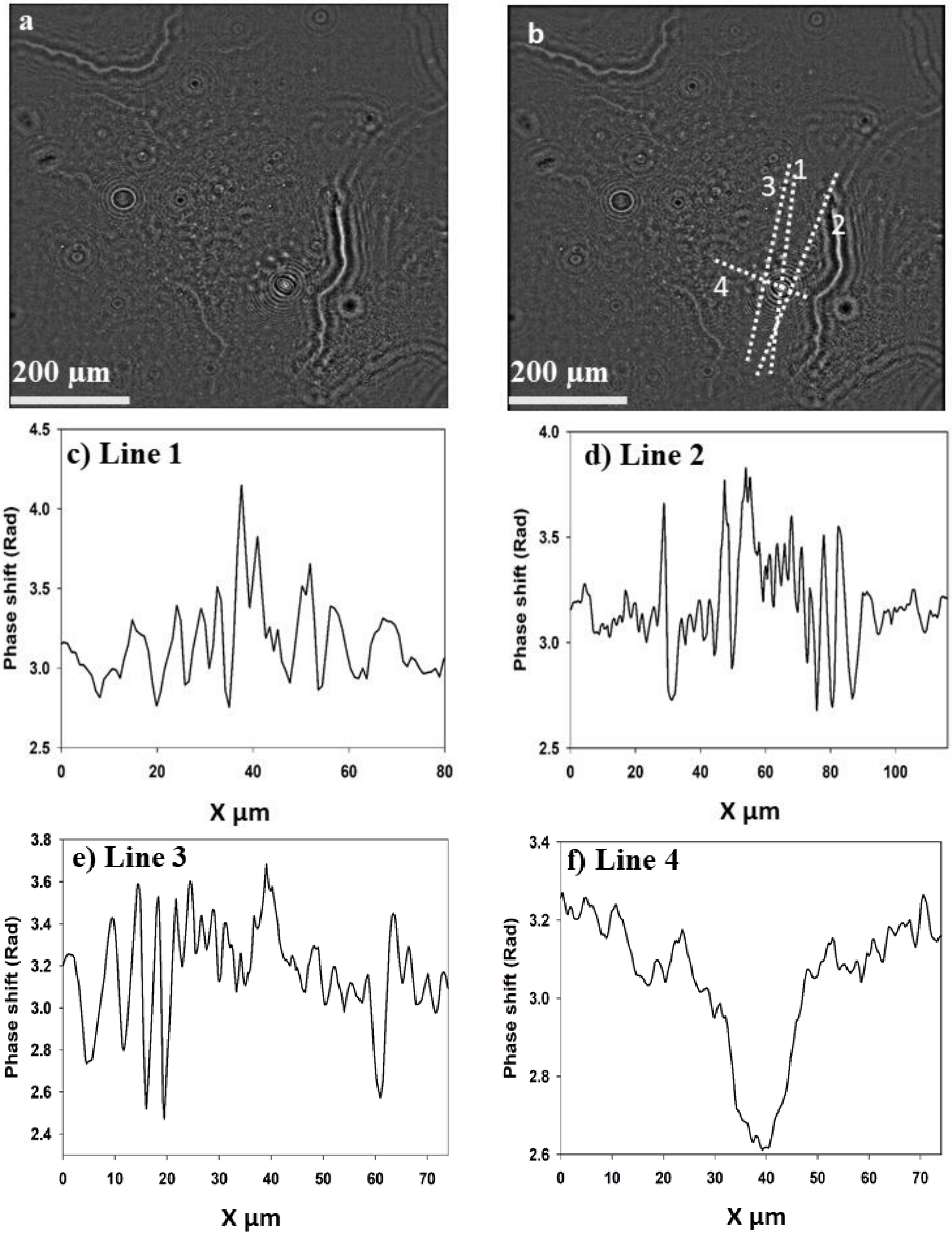

Figure S8 Phase image. (a, b) Phase reconstruction of particles in ambient air. (c-f) Phase profiles of particles through a crosscut across Lines 1 to 4.

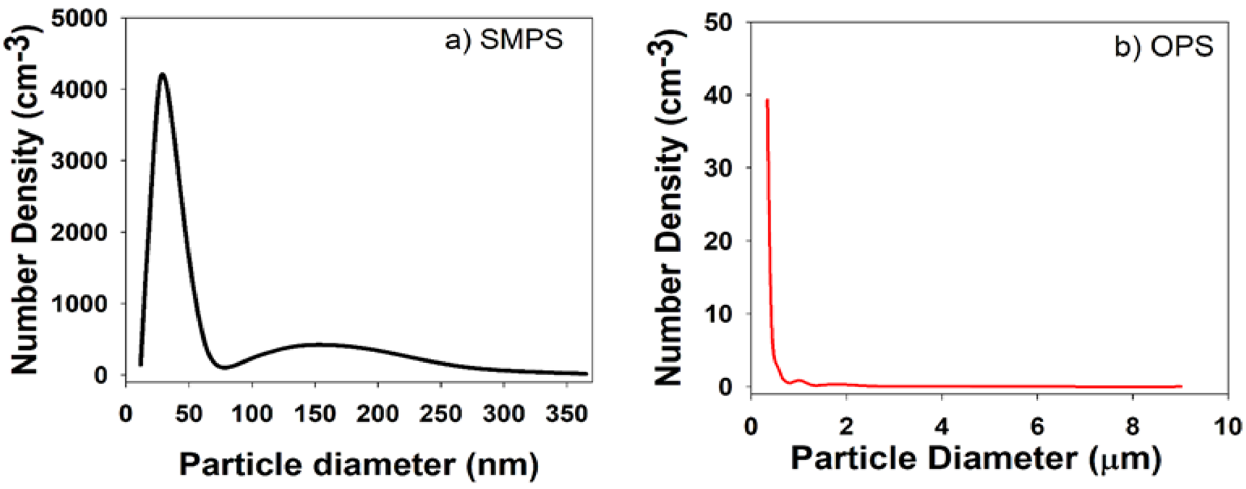

Figure S9 Particle size distribution of ambient air (a) measured by a scanning mobility particle sizer (SMPS) in the range from 10 nm to 350 nm. (b) measured by an optical particle sizer (OPS) in the range from 0.3 μm to 10 μm,.

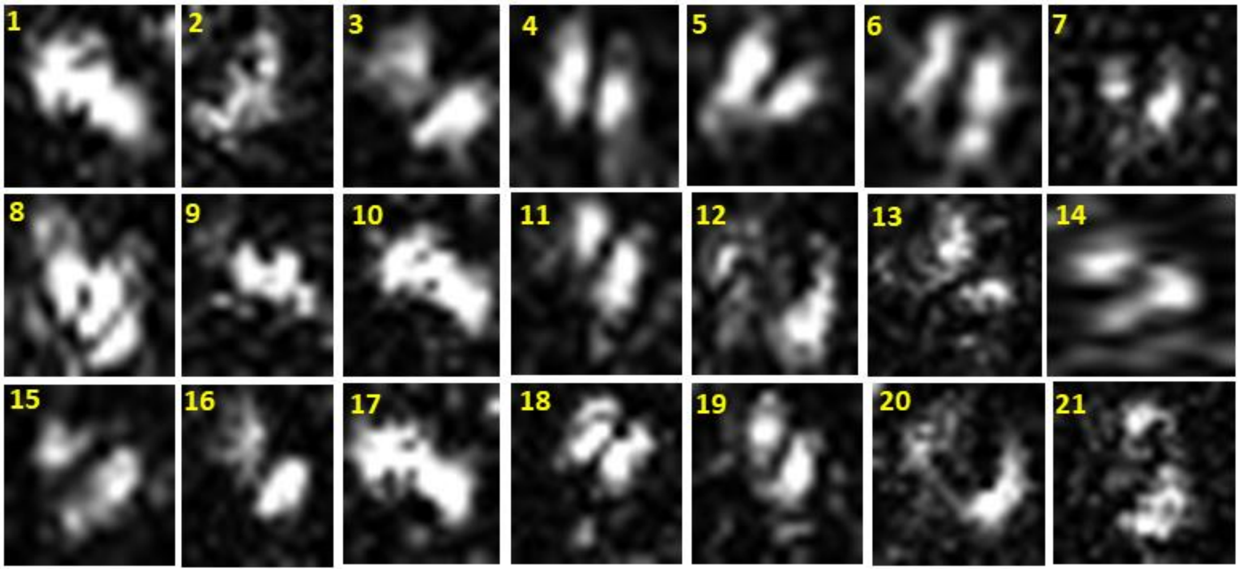

Objec	Object ima	X(μm)	Y(μm)	Z(μm)	Length(μm)	Width(μm)	Contour	Area(μm²)	Perimeter(μ	Edge gradient	Roughness	R1	R2	R3	R4	R5	SD	Hu1	Hu2	Hu3	Hu4	Hu5	Hu6	Hu7	Histogram
1		581.52	1202	5750	11.7585	10.0787		73.4337	34.443	130.223	1.06381	0.26	0.85	0.85	0.77	0.43	80.7	0.001	5.9E-09	6E-12	4.7E-11	-7E-22	-2.9E-15	3.1E-22	
2		828.25	985	5690	8.37751	4.56955		22.0408	20.6049	168.286	1.01973	0.36	0.48	0.6	0.48	0.48	83	0.001	1.7E-07	7E-11	3E-12	-3E-23	-1.1E-16	-2.9E-23	
3		962.72	883.1	4977	8.47796	7.53596		37.2689	25.3018	135.208	1.00624	0.71	0.94	0.94	0.83	0.59	98.8	9E-04	8.1E-08	2E-11	2.8E-12	1.6E-23	7.4E-16	8.5E-24	

Figure S10 A simple example of fast and automated reconstruction by stingray software of large volume of images, which were produced in xls file and individual images part of it is shown here.Automated detection of airborne 100 nm PSL particles by using stingray software. (a) As an example from number 1 to number 21 and so on shows the object Id, and (b) Exported results in xls format with the numerical values. Excel data sheet carried the information of particle orientation, particle size, perimeter, area, roughness and other morphology component of particles, momentum and spectrum of particles.

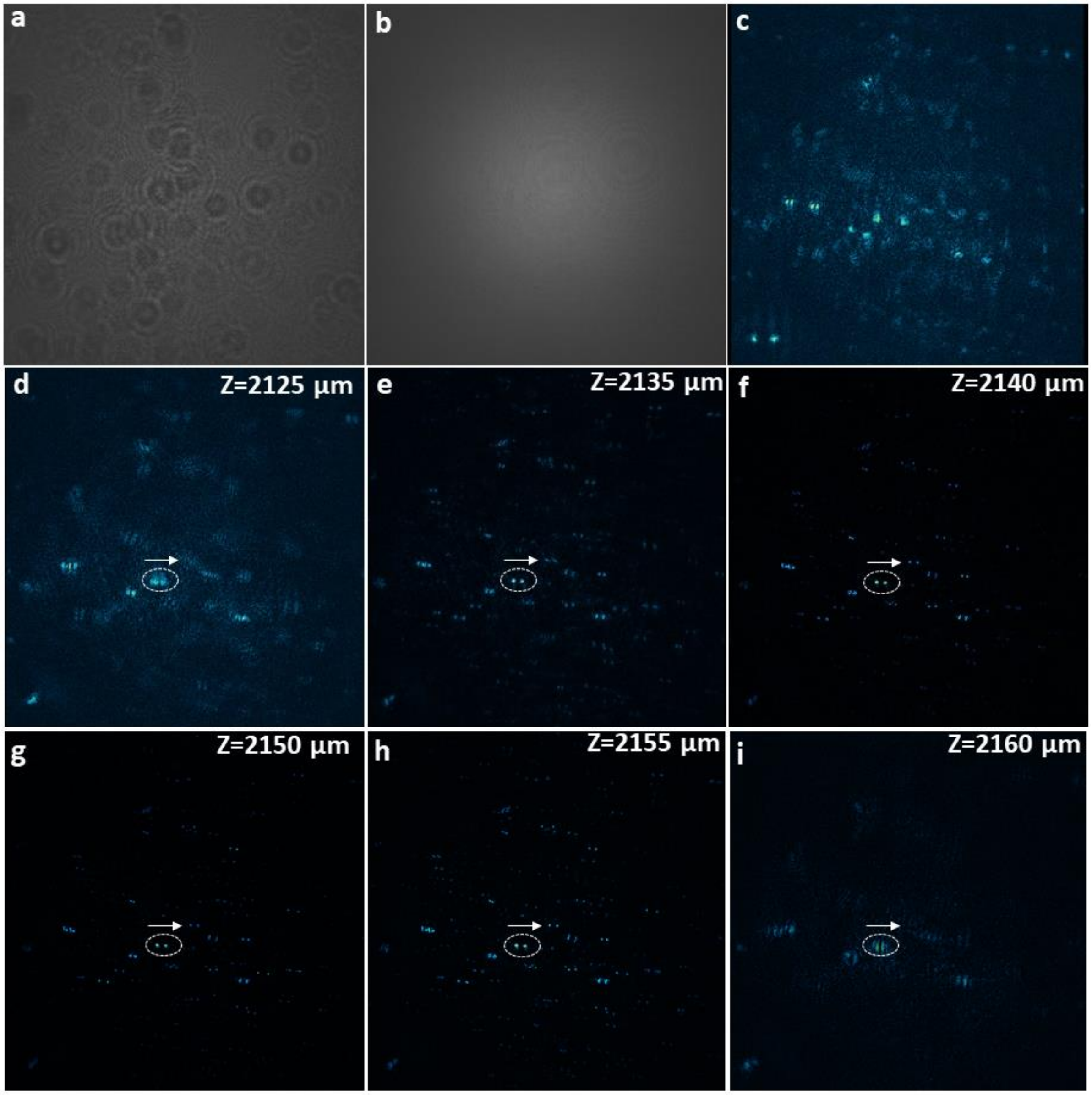

Figure S11 Intensity reconstruction at different depths of PSL particles on a microscopy slide. (a) Raw hologram recorded with PSL particles; (b) Background hologram recorded

without PSL particles; (c) Contrast hologram obtained after subtracting the background hologram from the raw hologram.(d-i) Reconstruction of particles at different reconstruction position Z. It shows the small changes in Z position changes the particles from focus to defocused.

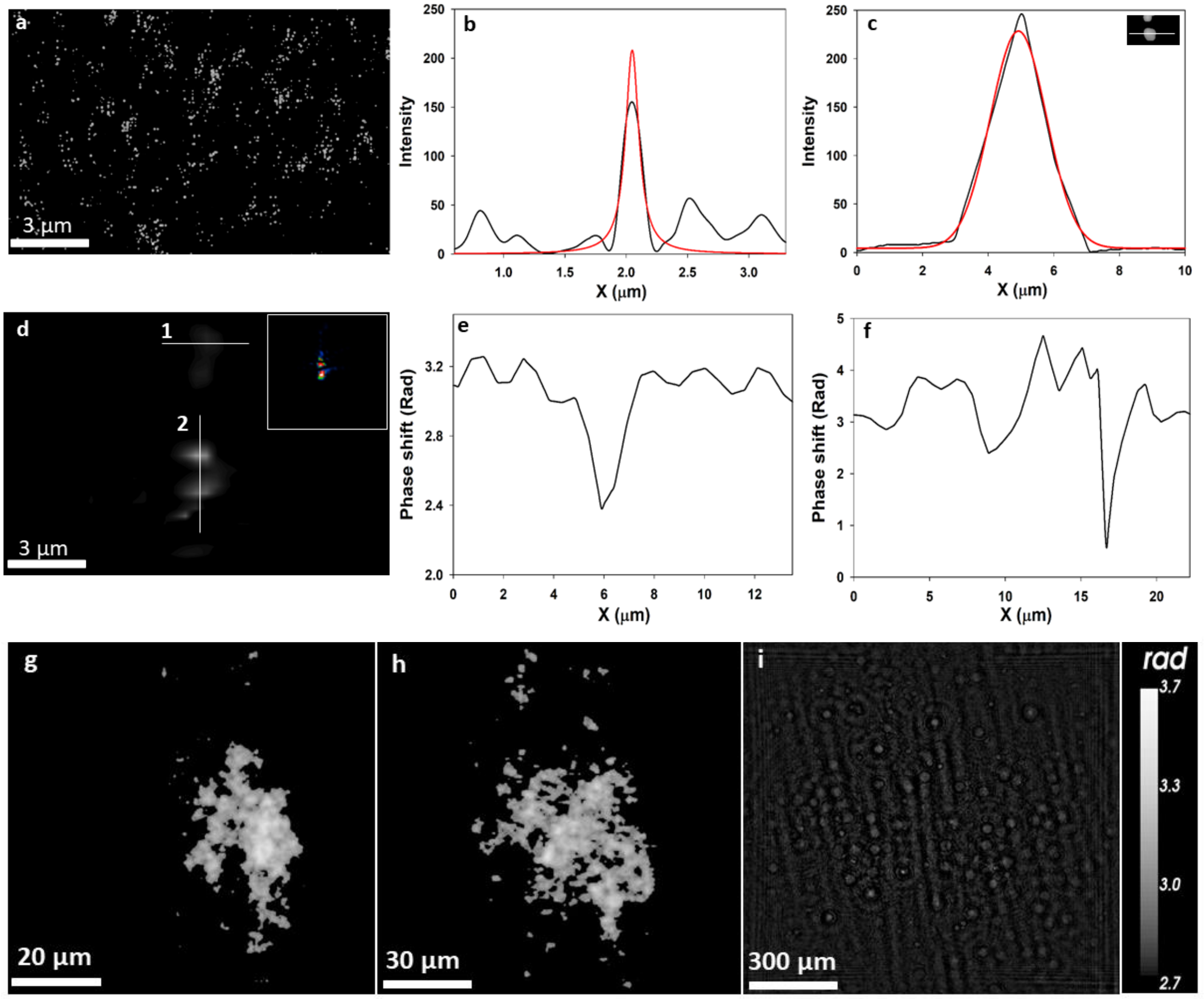

Figure S12 Hologram reconstruction for aerosolized snow meltwater. (a) Intensity reconstruction of particles. (b, c) Intensity profile through the crosscut of particles. (d) Phase reconstruction of particles. (e, f) Phase shift across the crosscut of particles through the line 1 and line 2. (g-i) Intensity reconstructions of particles in the snow meltwater in the liquid phase. The image in the selected area in (d) shows the same particles' intensity reconstruction. The red lines are the Gaussian quadratic fits for particles in (b, c).

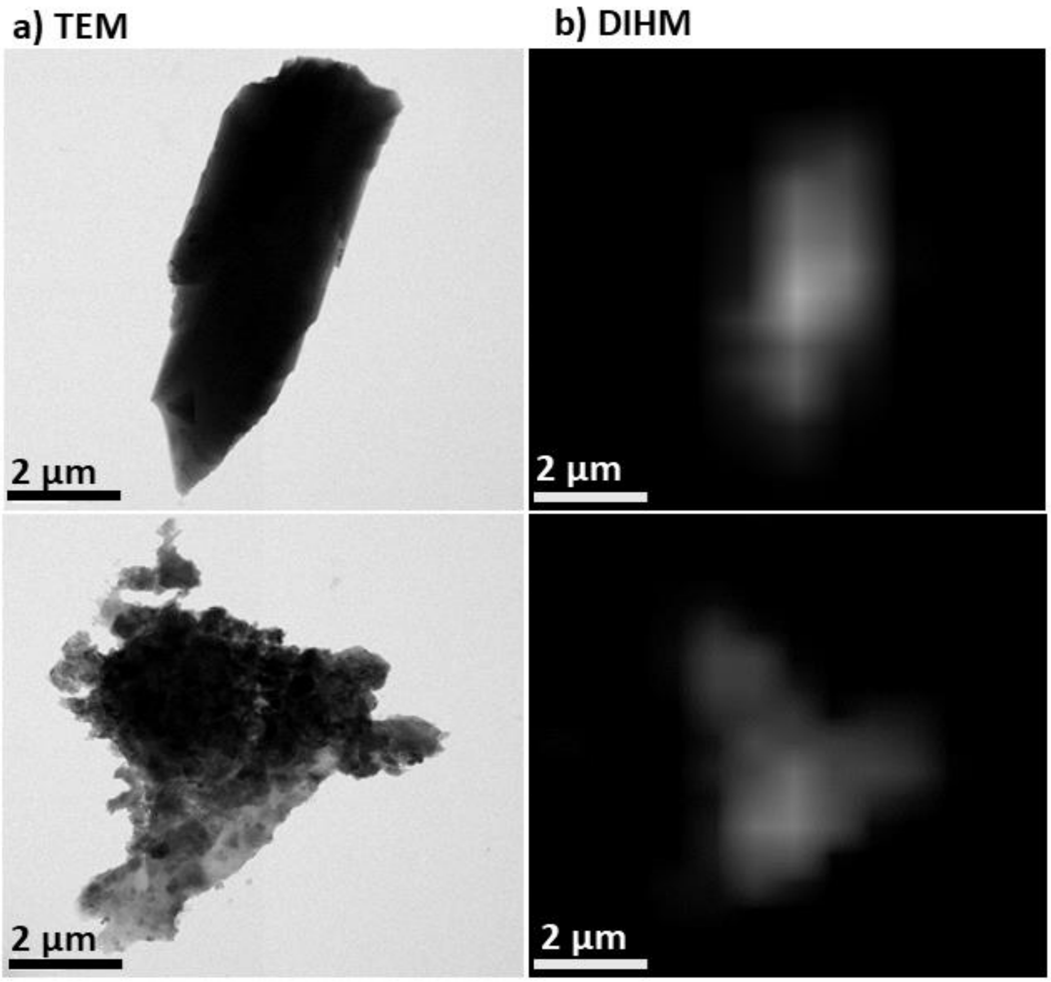

Figure S13 Morphology of snow meltwater in the liquid phase (a) visualized by STEM, and (b) visualized by Nano-DIHM.

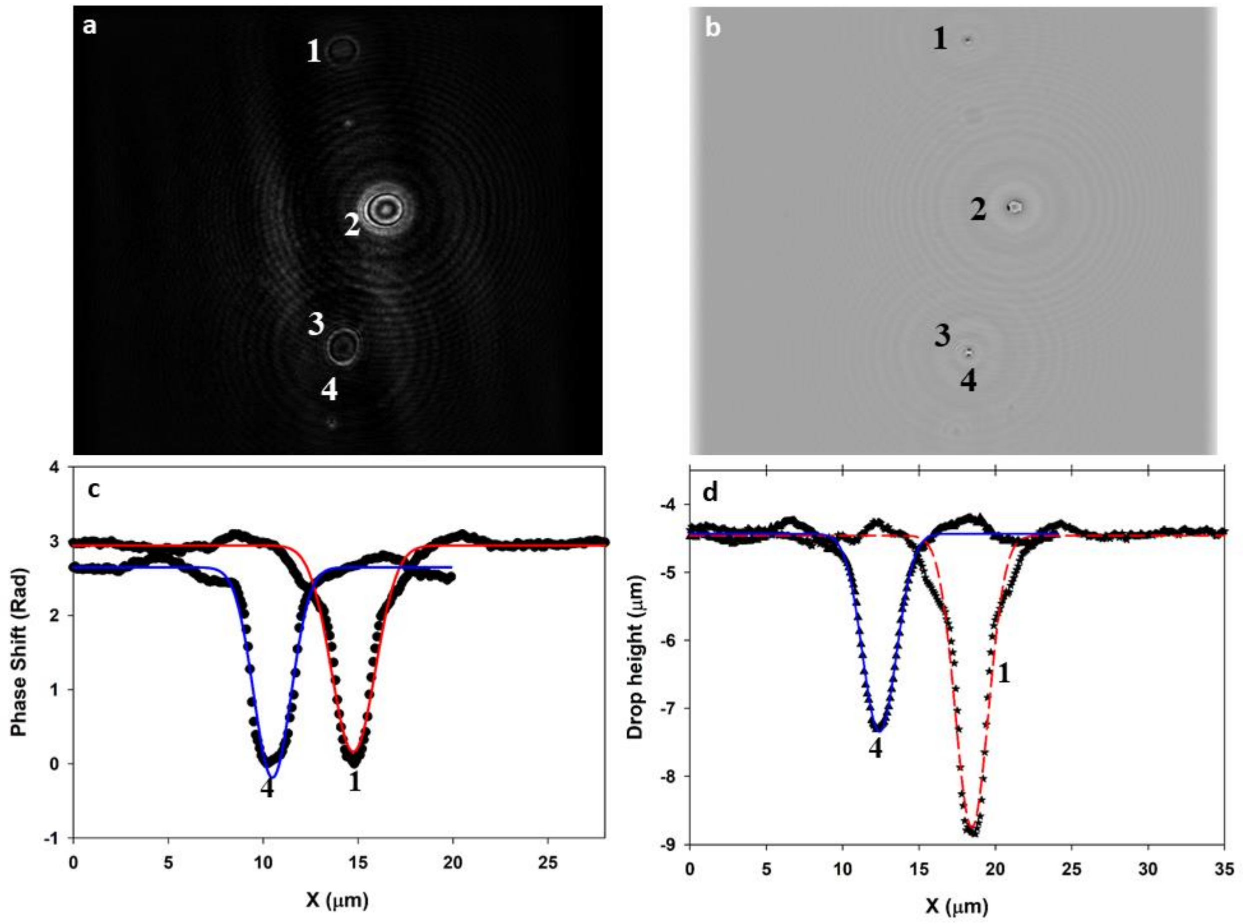

Figure S14 Glycerine drops in the suspension of type F microscope immersion oil. (a, b) Intensity and phase reconstruction of four glycerin drops in type F microscope oil. (c) Phase cuts through drops 1 and 4, and (d) drop height. The blue and red lines are the Gaussian quadratic fits.

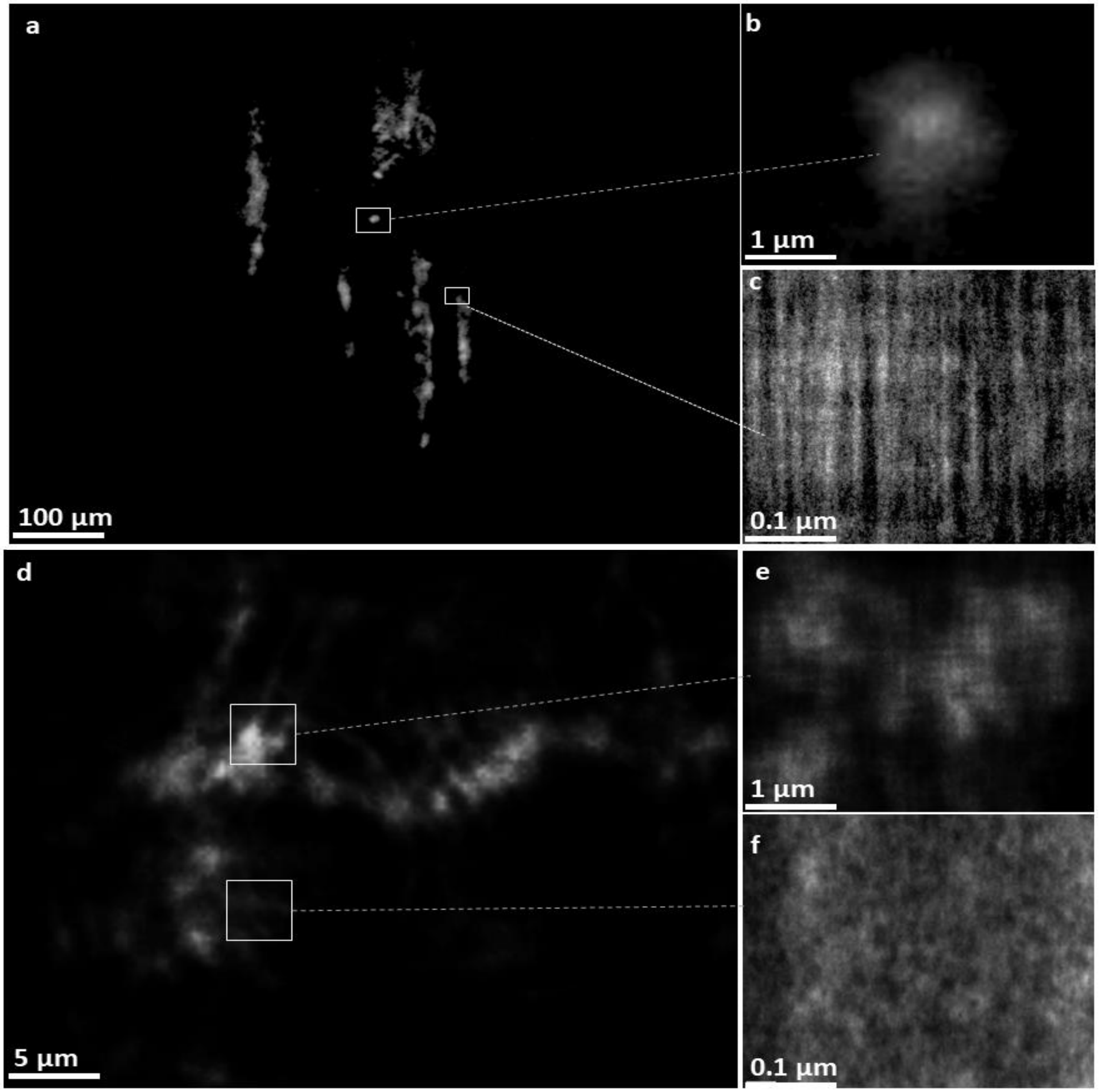

Figure S15 Shape, size and morphology of synthetic materials. (a) Iron oxide. (b, c) Iron oxide in the highlighted regions of Image (a). (d) Zinc oxide. (e, f) Zinc oxide in the box of image (d).

Supplementary Tables

Table S1 Descriptive statistics of the distributions of dimensions of the aerosolised Milli-Q water in 3D space in a single hologram.

Statistics	Width (µm)	Height (µm)	Length (µm)
**Mean values**	0.093	0.089	0.554
**Standard deviation**	0.195	0.170	1.060
**Median values**	0.041	0.043	0.273
**99th percentile**	0.785	0.700	3.873
**1st percentile**	0.010	0.010	0.062

Table S2 Descriptive statistics of the distributions of dimensions of the 100 nm PSL spheres, 200 nm PSL spheres deposited on the microscopy slide in 3D space in a single hologram.

**100 nm PSL**			
Statistics	Width (µm)	Height (µm)	Length (µm)
**Mean values**	0.158	0.177	0.765
**Standard deviation**	0.237	0.284	0.638
**Median values**	0.107	0.120	0.732
**99th percentile**	1.096	1.068	3.874
**1st percentile**	0.012	0.012	0.021
**200 nm PSL**			
**Mean values**	0.226	0.228	0.767
**Standard deviation**	0.220	0.219	0.840
**Median values**	0.172	0.154	0.588

**99th percentile**	0.947	1.110	3.887
**1st percentile**	0.013	0.012	0.015

Table S3 Changing refractive indices for different sizes of glycerine drops. Comparison of glycerin drop size as determined from phase shifts and from intensity reconstructions.

Drops*	Maximum phase shift (Rad)	Drop height from phase shift (µm)	Drop diameter from intensity image (µm)	Calculated change of refractive index (Δn)
1	2.8	4.15	4.0	0.0451
2	0.94	1.5	1.4	0.0433
3	2.3	3.3	3.2	0.0449
4	2.35	4.0	3.4	0.0446

*$\Delta n = (n_{glyc} - n_{oil}) = 0.0451$. Changing refractive index between glycerine and type F microscope immersion oil.

Supplementary Notes

Supplementary Note 1: Analysis of the size and phase of snow-borne particles in dynamic and stationary modes

We recovered the size and morphology of snow-borne particles in the gas and liquid phase. Figure S12 (a) presents the distribution of particles in the snow meltwater following aerosolization. Figure S12 (b, c) shows the intensity profile along with the particle crosscut. The intensity profiles confirmed that particles' sizes are 180 nm and 2.7 µm, respectively (Figure S12 (b, c)). The simultaneous size distribution measurements of the same samples made by the SMPS (Figure S5 (b)) confirmed the particle size of 180 nm, and the OPS data (Figure S5 (d)) confirmed the particle size of 2.7 µm.

The phase reconstruction of snow-borne particles in the gas phase across line 1 and line 2 is shown in Figure S12 (d). The quantitative phase shift varies from 2.3 to 3.3 rad (mean value = 3.04 rad) across line 1 (Figure S12 (e)). Across line 2, the phase shift shows multimodal variances from 0.6 to 4.7 rad (mean value = 3.3 rad) (Figure S12 (f)). The relationship between phase shift and refractive index (see methods) shows that the refractive index changes by 0.016 across line 1 and from 0.045 to 0.075 across line 2, suggesting that the particles are of polymeric origin [1,2].

To evaluate whether DIHM can determine various shapes, size, and morphology of individual particles, we investigated the same snow meltwater samples using both DIHM (Figure S12 (g, h) and Figure S13 (b)) and STEM (Figure S13 (a)). STEM images illustrate the non-spherical morphology of particles and agglomerates (Figure S13 (a)) matching the shapes visualized by Nano-DIHM (Figure S13 (b)) in the same snow meltwater samples.

Supplementary Note 2: Refractive index measurement

We determined the changing refractive indices of glycerine drops of different sizes suspended in type F microscope immersion oil. The intensity and phase reconstruction of several glycerin drops suspended in type F microscope oil are shown in Figure S14 (a, b). Two examples of the phase variation through drops 1 and 4 were examined (Figure S14 (c)). Figure S14 (d) shows the drop height for drops 1 and 4. The phase examined for glycerine drops showed almost noise-free cross-sections except for a small phase variation of the background (Figure S14 (c)).

From the known refractive index difference between glycerin and type F microscope oil ($\Delta n=0.0451$) and the measured maximum phase shifts for a drop, we can calculate the drop height or vice versa (see Methods)[3]. The quantitative phase, size, and refractive index measurements for the four glycerine drops shown in Figure S14 (a, b) are also presented in Table S3. Table S3 shows the changing refractive index between 0.0443 to 0.0451 and drop heights between 1.5 to 4.15 μm. The determination of the changing refractive index of each drop size varies within ~ 2%. In comparison, the agreement between the drop height calculated from the maximum phase shift and the diameter obtained from intensity reconstructions was observed within ∼10%. The small variation in measured refractive index or drop height could be related to substrate adhesion.

Supplementary Note 3: Synthetic materials

The shape, size, and morphology of synthetic materials (zinc oxide and iron oxide) have been successfully determined using DIHM. Figure S15 (a) shows the reconstructed image of iron oxide powder over the microscope cover slide. Higher resolution images in Figure S15 (b, c) depict the different sizes of iron oxide particles. Similarly, different sizes of zinc oxide particles were determined using DIHM (Figure S15 (d-f)). DIHM showed the morphology of the synthetic materials and the presence of nanoparticles.

Supplementary Note 4: Automation of stingray software

An essential stingray software has been bought with the 4deep desktop holography microscope[1,2]. The octopus software[2] has been used manually to reconstruct the hologram, while stingray software can be automated to detect the objects for millions of holograms. Stingray can be used to analyze holograms both in real-time or offline. The first input is to train the stingray software by providing the recording conditions of holograms (hologram size, camera pixel, source to sensor distance); the second is to optimize the intensity of the holograms or find the threshold intensity values. We used a grayscale and maximum intensity with 250 arbitrary units.

Based on the threshold value of intensity, Stingray will start looking to detect the objects. The main issue is to identify the threshold values of intensity. For that, we used octopus software for hundreds of hologram reconstructions and estimated the best threshold values. Here are some consequences if we choose high or low threshold values, such as a high threshold, for example, 200, which means that the pixel's intensity has to be 200 or more for that pixel to be detected by the software. Thus, a low threshold (ex: 10) would allow many pixels to be detected, while a high threshold (ex: 200), would let fewer pixels be detected. For that, we started with a low threshold (for example, 50) and worked our way up to a higher threshold until we find optimized conditions. The major advantage of fully automated Stingray software is to distinguish the round vs randon shaped particles along with time dependent morphology, roughness and orientation of objects. However, the size obtained by stringray software required further correction. Currently, we are in the process of building the library for different kinds of samples and estabishing the size correction methods (Manuscript in under preparation).

Supplementary References

1. Kudo, H., Yamamoto, M., Nishikubo, T. & Moriya, O. Novel Materials for Large Change in Refractive Index: Synthesis and Photochemical Reaction of the Ladderlike Poly(silsesquioxane) Containing Norbornadiene, Azobenzene, and Anthracene Groups in the Side Chains. *Macromolecules* **39**, 1759-1765, doi:10.1021/ma052147m (2006).
2. Hart, S. J. & Terray, A. V. Refractive-index-driven separation of colloidal polymer particles using optical chromatography. *Applied Physics Letters* **83**, 5316-5318 (2003).
3. Jericho, M. H., Kreuzer, H. J., Kanka, M. & Riesenberg, R. Quantitative phase and refractive index measurements with point-source digital in-line holographic microscopy. *Applied Optics* **51**, 1503-1515, doi:10.1364/AO.51.001503 (2012).
