# Supplementary material for: Advancing the science of dynamic airborne nanosized particles using Nano-DIHM
Source: Commun Chem. 2021 Dec 8;4:170. doi: 10.1038/s42004-021-00609-9 (PMC9814397; doi:10.1038/s42004-021-00609-9)
Supplement: Supplementary file 3 — Description of Additional Supplementary Files [file 42004_2021_609_MOESM3_ESM.pdf]

Description of Additional Supplementary Files

File Name: Supplementary Movie 1

Description: The dynamic 4D trajectories (3D positions and 1D time) of 100 nm PSL spheres are provided in Figure 4 (a-d) in the main text. The movie-SI-Giff 1 is a zoomed-in image of Supplementary Movie 2.

File Name: Supplementary Movie 2

Description: The dynamic 4D trajectories (3D positions and 1D time) of 100 nm PSL spheres for a long time. The particle trajectories are reconstructed from the time-dependent experimental images (supplementary Movie 1) with a temporal resolution of 62.5 ms.
