# Supplementary figures and images for: Advancing the science of dynamic airborne nanosized particles using Nano-DIHM
Source: Commun Chem. 2021 Dec 8;4:170. doi: 10.1038/s42004-021-00609-9 (PMC9814397; doi:10.1038/s42004-021-00609-9)

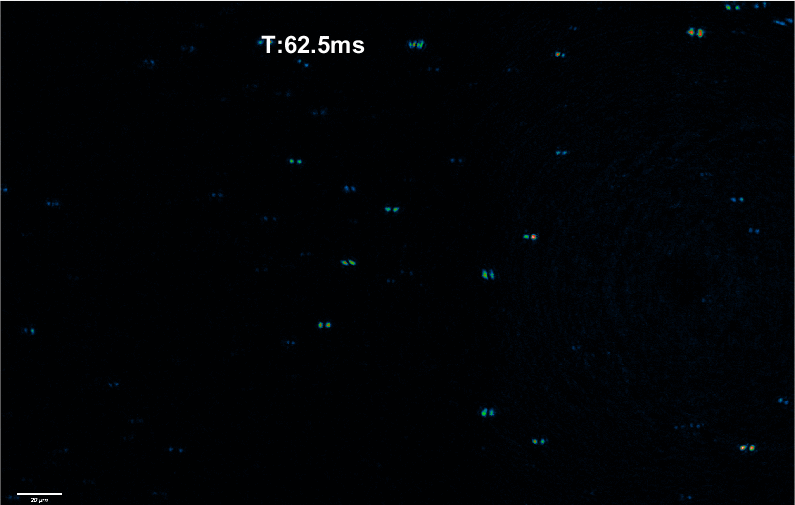

Supplement: Supplementary file 4 — Supplementary Movie 1 [file 42004_2021_609_MOESM4_ESM.gif]

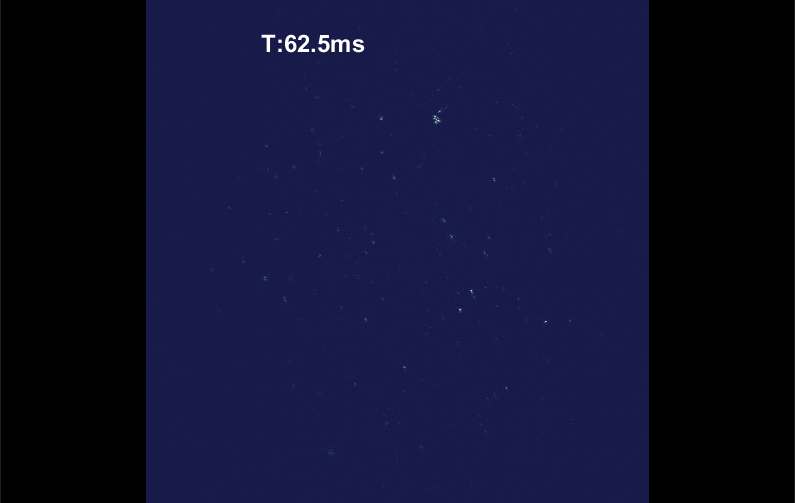

Supplement: Supplementary file 5 — Supplementary Movie 2 [file 42004_2021_609_MOESM5_ESM.gif]
